# Compensation and Aid for Air-Raid Victims

**Published:** 1918-02-09

**Authors:** 


### COMPENSATION AND AID FOR AIR-RAID VICTIMS.

BY A SOCIAL WORKER.

It is to be hoped that the Government arrangements for compensation to air-raid victims and for their immediate care have become widely known and understood. It will certainly be well if all Councils will notify, as has in some places been done by posters, the proper places at which to apply. In addition to the unavoidable agitation and danger of the actual raid time, anxiety as to the possible suffering by children and uncompensated loss of the few and cherished possessions of the poor is an intolerable burden. It has fallen heavily upon some wives left alone now in charge of family and home, and already sufficiently strained by uncertainty as to the fate of husband and perhaps of sons. Imagine such a mother, so shaken in nerves that when daylight fades the house becomes unendurable, dressing several children, the youngest but two years old, and going forth to walk the streets till near midnight, afraid even to face the crowd and airlessness of the Tubes. Imagine, too, a child so distraught by the remembrance of scenes beheld that the mere sound of some small object falling on the floor sends her into a dazed condition that may last for an hour. Such cases are actually among those which have been cared for by Miss Gardiner under the excellent little experimental scheme which has taken a number of mothers and children to convalesce at a country place in the Midlands. It is evident that without some such relief a serious mental or physical breakdown threatens, and more of such kindly and practical schemes are greatly to be desired.

### No Need for Fresh Machinery.

The important point in actual relief is instant aid. *Bis dat qui cito dat.* Probably neighbours will open their doors willingly to the refugees, but many such hospitable roofs in the poorer quarters have the wolf never very far from the door, and it is imperative that the actual replacement of loss in things needed for daily use should be prompt and maintenance available. There should be no need to set up fresh machinery, beyond the cards actually required to be filled up for registration and verification of claims. If centred in the Councils as deputies of the Local Government Board, aid can be entrusted to recognised organisations, in the same way that War Pensions work has been handed over to the already existing S.S.F.A. Committees, so that voluntary yet experienced agents can deal with the cases in detail. In this way advances can be immediately made, while claims for grants are investigated. It is a wise provision that authorises local committees to remove and store furniture and help to re-house the family, especially as consequential loss or theft is, necessarily perhaps, ruled out from compensation, and also fees incurred in "preparing or supporting a claim on the Government."

### The Government Scheme.

The Government compensation scheme applies to property in the United Kingdom of aggregate value not above £500, and took effect as from September 1, 1917, so that all losses from air raid will be included, whether or not the property was insured under the Government scheme. Property otherwise insured will of course not be affected, and a claim found fraudulent in any respect will be liable to total rejection. Compensation will not be made for loss of "money, securities, stamps, documents, MSS., books of account." The safe keeping of such things as savings-bank books or war-saving certificates is often a difficulty to the really poor, even to save them from children's fingers. One remarkable result of the present high wages is the large quantity of household furniture bought, and the result in greater orderliness and consequent house-pride for the mother promises to be a real gain, but this now increases the risk of loss. Friends of the poor may well suggest to them now the keeping of valuables in a bag that could easily be taken to a safe place.

Damage to life and limb will be met by payment of medical expenses and appliances, funeral expenses paid up to £9. Temporary maintenance grants may be made up to the equivalent of the Army separation allowances, supplemented in very special cases. We must hope that the first cases judged on their merits will be dealt with in a liberal spirit. Whether or no the bombing of working-class districts is an intentional effort by the enemy to foster pacifism, it is certain that some bitterness and jealousy of classes less poignantly affected may easily arise.

No time should be lost in forming committees where that has not yet been done. To them funds for relief of distress, as distinguished from compensation, will be entrusted. They will investigate and forward claims for uninsured property to the Air-Raid Compensation Committee (Palmerston House, Old Broad Street, E.C. 2), and are desired to keep a list of lodgings or shelters available. If Poor-Law Institutions are used it is expressly noted that the sufferers are to be treated as guests, to avoid all suggestion of disgrace.

